# Epigenetic Drift Association with Cancer Risk and Survival, and Modification by Sex

**DOI:** 10.3390/cancers13081881

**Published:** 2021-04-14

**Authors:** Chenglong Yu, Ee Ming Wong, Jihoon Eric Joo, Allison M. Hodge, Enes Makalic, Daniel Schmidt, Daniel D. Buchanan, Gianluca Severi, John L. Hopper, Dallas R. English, Graham G. Giles, Melissa C. Southey, Pierre-Antoine Dugué

**Affiliations:** 1 Precision Medicine, School of Clinical Sciences at Monash Health, Monash University, Clayton, VIC 3168, Australia; chenglong.yu@monash.edu (C.Y.); eeming.wong@monash.edu (E.M.W.); graham.giles@cancervic.org.au (G.G.G.); melissa.southey@monash.edu (M.C.S.); 2 Department of Clinical Pathology, Melbourne Medical School, The University of Melbourne, Parkville, VIC 3010, Australia; 3 Colorectal Oncogenomics Group, Department of Clinical Pathology, Melbourne Medical School, The University of Melbourne, Parkville, VIC 3010, Australia; ji.joo@unimelb.edu.au (J.E.J.); daniel.buchanan@unimelb.edu.au (D.D.B.); 4 University of Melbourne Centre for Cancer Research, Victorian Comprehensive Cancer Centre, Parkville, VIC 3010, Australia; 5 Cancer Epidemiology Division, Cancer Council Victoria, Melbourne, VIC 3004, Australia; allison.hodge@cancervic.org.au (A.M.H.); dallas.english@cancervic.org.au (D.R.E.); 6 Centre for Epidemiology and Biostatistics, Melbourne School of Population and Global Health, The University of Melbourne, Parkville, VIC 3010, Australia; emakalic@unimelb.edu.au (E.M.); Daniel.Schmidt@monash.edu (D.S.); j.hopper@unimelb.edu.au (J.L.H.); 7 Genomic Medicine and Family Cancer Clinic, Royal Melbourne Hospital, Parkville, VIC 3000, Australia; 8 Centre de Recherche en Epidémiologie et Santé des Populations (CESP, Inserm U1018), Facultés de Médecine Universités Paris-Saclay, UVSQ, Gustave Roussy, 94805 Villejuif, France; gianluca.severi@inserm.fr; 9 Department of Statistics, Computer Science and Applications “G. Parenti”, University of Florence, 50121 Firenze, Italy

**Keywords:** ageing, sex difference, age-by-sex, cancer risk, cancer survival, DNA methylation, pre-diagnostic blood, X chromosome, epigenetic drift

## Abstract

**Simple Summary:**

Ageing is the strongest cancer risk factor, and men and women exhibit different risk profiles in terms of incidence and survival. DNA methylation is known to strongly vary by age and sex. Epigenetic drift refers to age-related DNA methylation changes and the tendency for increasing discordance between epigenomes over time, but it remains unknown to what extent the epigenetic drift contributes to cancer risk and survival. The aims of this study were to identify age-associated, sex-associated and sexually dimorphic age-associated (age-by-sex-associated) DNA methylation markers and investigate whether age- and age-by-sex-associated markers are associated with cancer risk and survival. Our study, which used a total of 2754 matched case–control pairs with DNA methylation in pre-diagnostic blood, is the first large study to examine the association between sex-specific epigenetic drift and cancer development and progression. The results may be useful for cancer early diagnosis and prediction of prognosis.

**Abstract:**

To investigate age- and sex-specific DNA methylation alterations related to cancer risk and survival, we used matched case–control studies of colorectal (*n* = 835), gastric (*n* = 170), kidney (*n* = 143), lung (*n* = 332), prostate (*n* = 869) and urothelial (*n* = 428) cancers, and mature B-cell lymphoma (*n* = 438). Linear mixed-effects models were conducted to identify age-, sex- and age-by-sex-associated methylation markers using a discovery (controls)-replication (cases) strategy. Replication was further examined using summary statistics from Generation Scotland (GS). Associations between replicated markers and risk of and survival from cancer were assessed using conditional logistic regression and Cox models (hazard ratios (HR)), respectively. We found 32,659, 23,141 and 48 CpGs with replicated associations for age, sex and age-by-sex, respectively. The replication rates for these CpGs using GS summary data were 94%, 86% and 91%, respectively. Significant associations for cancer risk and survival were identified at some individual age-related CpGs. Opposite to previous findings using epigenetic clocks, there was a strong negative trend in the association between epigenetic drift and risk of colorectal cancer. Methylation at two CpGs overlapping *TMEM49* and *ARX* genes was associated with survival of overall (HR = 0.91, *p* = 7.7 × 10^−4^) and colorectal (HR = 1.52, *p* = 1.8 × 10^−4^) cancer, respectively, with significant age-by-sex interaction. Our results may provide markers for cancer early detection and prognosis prediction.

## 1. Introduction

Ageing is the strongest risk factor for cancer overall, and for the majority of individual cancer types [1]. Advanced age is typically associated with increased cancer risk and reduced cancer survival [2,3,4]. Males and females exhibit different risk profiles in terms of incidence and survival [5,6,7,8,9,10], and most cancers for which there is a clear sex difference affect men more frequently than women, with incidence rates ranging from 1.3:1 for Hodgkin lymphoma to 4.9:1 for oropharynx and tonsil cancer [10]. Several studies [11,12,13,14,15] have investigated age- and sex-specific incidence and mortality rates using population-based data. However, the mechanisms underlying the observed age-by-sex interplay are not fully understood. As DNA methylation pattern is known to strongly vary by age [16,17] and sex [18,19], a key question of interest is whether DNA methylation alterations, and in which genes, show sex-specific differences during the ageing process and whether these are associated with development and progression of cancer.

Epigenetic drift is the tendency for increasing discordance between epigenomes over the lifetime [20]. Larger and higher genome-coverage studies have confirmed that the DNA methylation landscape of normal cells changes substantially with age [21], but it remains unknown to what extent the epigenetic drift contributes to cancer risk and survival. Many authors, including Horvath and colleagues [22,23,24], have investigated correlations between DNA methylation and ageing, and found that males have increased DNA methylation-based age acceleration (i.e., a “faster ticking” epigenetic clock) relative to females [25]. This finding was consistently reproduced in other studies [26,27]. We and others previously found that these epigenetic aging measures were associated with risk of and survival from specific types of cancers, but the contribution of the epigenetic drift as a whole has received less attention [28,29,30,31,32]. A recent study [33] has identified genome-wide methylation sites at which there was chronological age-by-sex interaction and found that most of them were on the X chromosome. To the best of our knowledge; however, no study has specifically investigated whether sex-specific DNA methylation alterations related to aging are associated with cancer risk and survival.

In this study, our aims were (i) to identify age-associated and sex-associated DNA methylation markers; (ii) to identify sexually dimorphic age-associated (age-by-sex-associated) DNA methylation markers; (iii) to investigate whether age- and age-by-sex-associated methylation markers are associated with cancer risk and survival.

## 2. Results

### 2.1. Characteristics of Study Samples

The characteristics of the Melbourne Collaborative Cohort Study (MCCS) samples used in this study are presented in Table 1 and Table S1. Controls were matched to cases on age at blood draw, sex, country of birth (Australia/New Zealand, Greece, Italy, and UK/other) and sample type (peripheral blood mononuclear cells, dried blood spots and buffy coats). For the lung cancer study, controls were also matched on smoking history at the time of blood collection. Compared with controls, cases were more frequently past and current smokers, and had greater smoking pack-years (Table 1). The characteristics of the samples for each of the seven individual cancer case–control studies (colorectal, gastric, kidney, lung, prostate and urothelial cancers, and mature B-cell lymphoma) nested within the MCCS are described in Table S1.

### 2.2. Identification of Age-, Sex- and Age-by-Sex-Associated Methylation CpGs

**Figure 1:**
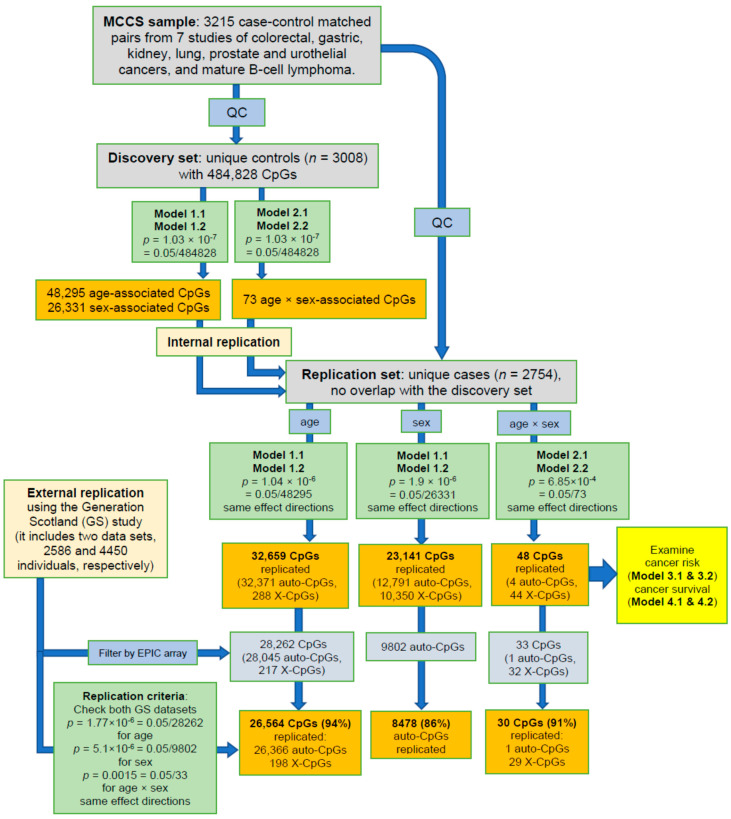
Flowchart of the discovery and replication strategy of the study. GS: Generation Scotland study. Auto-CpG and X-CpG: autosomal and X-linked CpGs, respectively. The GS study reported sex-associated results for autosomal sites only [33]. For model details, see Materials and Methods.

**Figure 2:**
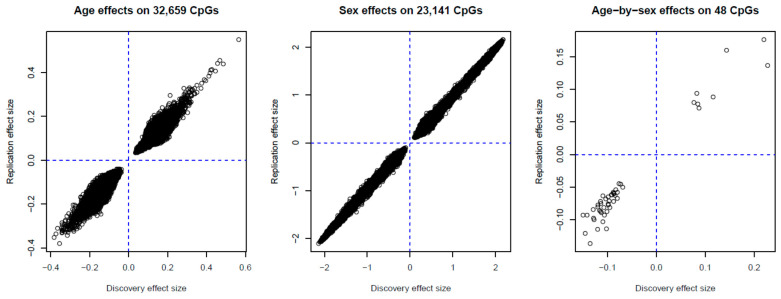
Regression coefficients in MCCS discovery and replication sets for the association between age, sex, and age-by-sex with blood DNA methylation.

The discovery phase used participants selected as controls in the methylation case–control studies and identified 48,295 age-associated and 26,331 sex-associated (*p* < 1.0 × 10^−7^) methylation sites in Models 1.1 and 1.2 (without and with adjustment for smoking, alcohol consumption and body mass index (BMI), respectively; for methodology details, see Materials and Methods, and Figure 1). We also detected 73 methylation sites at which there was a significant interaction (*p* < 1.0 × 10^−7^) between age and sex using Models 2.1 and 2.2 that included an age × sex interaction term. Among these markers, we replicated, using participants who were selected as cases: 32,659 age-associated CpGs (32,371 autosomal and 288 X-linked) (*p* < 1.0 × 10^−6^, 68%) (Table S2), 23,141 sex-associated CpGs (12,791 autosomal and 10,350 X-linked sites) (*p* < 1.9 × 10^−6^, 88%) (Table S3), and 48 age-by-sex-associated CpGs (4 autosomal and 44 X-linked sites) (*p* < 6.9 × 10^−4^, 66%) (Table S4). Regression coefficients of replicated associations for age, sex and age-by-sex in the discovery and replication sets are presented in Figure 2. Associations of methylation M-values for these 48 CpGs showing age-by-sex interaction are shown in Figure S1 (discovery set).

We further examined the replicated methylation markers using summary statistics results from two datasets from the Generation Scotland (GS) study [33]. We found that 94% age-associated (*p* < 1.8 × 10^−6^), 86% sex-associated (*p* < 5.1 × 10^−6^) and 91% age-by-sex-associated (*p* < 0.0015) CpGs were replicated in GS data (Figure 1). The 30 replicated age-by-sex methylation markers (1 autosomal and 29 X-linked sites) are shown in Table 2 and Table S4.

### 2.3. Cancer Risk

Of the 32,659 age-related methylation sites, two (cg25119261 in *HLA-DPB2* and cg05497216 in *ANKRD11*) were associated with risk of cancer overall (*p* < 1.5 × 10^−6^ = 0.05/32,659), and six (overlapping *AHSP*, *SBK1*, *RASGRP4* and *DPEP3* genes) were associated with risk of mature B-cell lymphoma (*p* < 1.5 × 10^−6^) (Table 3). These CpGs were all replicated as being associated with age in the GS data. Methylation M-values at these CpGs were both (1) all negatively associated with cancer risk (Table 3) and (2) all negatively associated with age (Table S2). We found no evidence to support that methylation at any of the 48 CpGs showing age-by-sex interaction was associated with risk of cancer overall or specific type (*p* > 0.001 = 0.05/48). A potential age-by-sex-related CpG, cg26738106 (chrX:3265038, TSS1500 of *MXRA5*), detected in the discovery set (beta = 0.09 and *p* = 3.0 × 10^−9^ in Model 2.2) was associated with colorectal cancer risk with OR = 0.58 (95% CI: 0.44–0.77), *p* = 0.0001 in Model 3.1, and OR = 0.59 (95% CI: 0.44–0.78), *p* = 0.0002 after adjustment for smoking, alcohol consumption and BMI (Model 3.2). However, this CpG was not replicated as showing age-by-sex-related methylation in the set of MCCS cases (beta = 0.01 and *p* = 0.36 in Model 2.2) and not included in the EPIC array used by McCartney et al. [33]’s GS study. 

**Figure 3:**
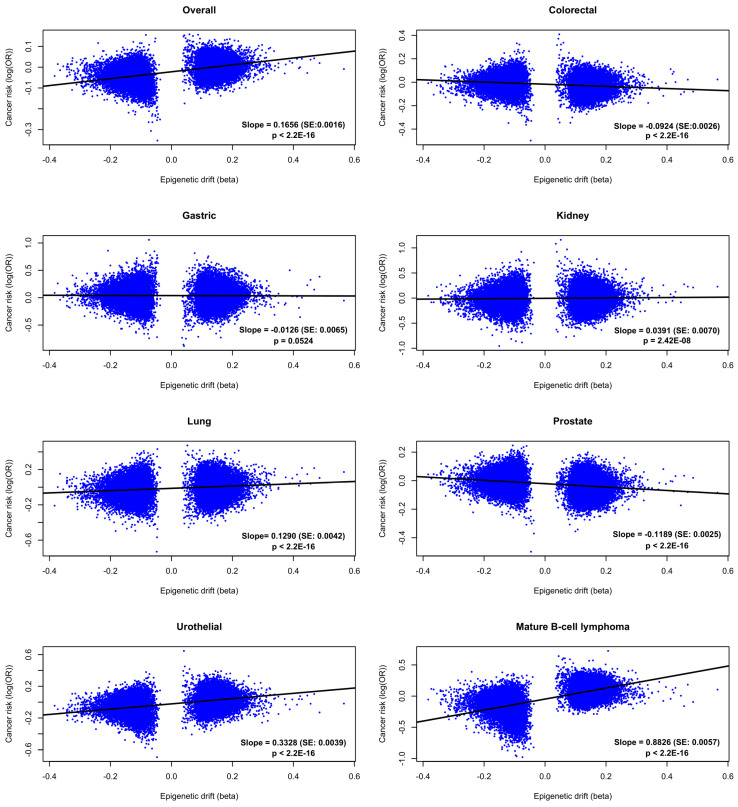
The trend in the association between epigenetic drift and risk of seven cancer types and overall. Linear regression Y~X was performed based on the 32,659 age-associated CpGs, where Y is the log (OR) for the association between DNA methylation and cancer risk and X is the regression coefficient (beta) for the association between DNA methylation and age. Slope of the regression line, standard error (SE) and *p*-value were obtained in the linear regression.

We further examined the trend of the association between epigenetic drift and risk of overall and specific cancers using the 32,659 age-associated CpGs. Figure 3 shows the correlations between the regression coefficients of the association between DNA methylation and age and the logarithm of ORs for the association between DNA methylation and cancer risk (the former was calculated in the discovery set). There was a strong positive correlation (beta = 0.17, *p* < 2.2 × 10^−16^) between associations of methylation with age and associations with overall cancer risk; the correlation was also strongly positive for kidney (beta = 0.04, *p* = 2.4 × 10^−8^), lung (beta = 0.13, *p* < 2.2 × 10^−16^) and urothelial (beta = 0.33, *p* < 2.2 × 10^−16^) cancers and mature B-cell lymphoma (beta = 0.88, *p* < 2.2 × 10^−16^); a negative correlation was observed for colorectal (beta = −0.09, *p* < 2.2 × 10^−16^) and prostate (beta = −0.12, *p* < 2.2 × 10^−16^) cancers.

### 2.4. Cancer Survival

Of the 32,659 age-related CpGs, 126 (all autosomal) were associated with survival of cancer overall (*p* < 1.5 × 10^−6^) (Table S5). The results for the 20 most significant associations are presented in Table 4. One age-by-sex-associated CpG cg12054453 (chr17:57915717) in the gene body of *TMEM49* was associated with survival of cancer overall (*p* < 0.001) with HR = 0.88 (95% CI: 0.83–0.93), *p* = 2.3 × 10^−6^ in Model 4.1 and HR = 0.91 (95% CI: 0.86–0.96), *p* = 7.7 × 10^−4^ after adjustment for lifestyle-related confounders (Model 4.2). 

We also examined the associations of age-related blood DNA methylation with survival for each individual cancer type and detected associations for gastric cancer (30 autosomal sites), lung cancer (282 autosomal and 1 X-linked sites) and mature B-cell lymphoma (1 autosomal site) (Table S6). There was one age-by-sex-related CpG (cg21729122) in the TSS1500 region of *ARX* (X chromosome) that was associated with survival of colorectal cancer with HR = 1.59 (95% CI: 1.28–1.97), *p* = 2.3 × 10^−5^ in Model 4.1 and HR = 1.52 (95% CI: 1.22–1.89), *p* = 1.8 × 10^−4^ in Model 4.2. There was no evidence to support an association of age-by-sex-associated methylation sites with survival of other cancer types.

## 3. Discussion

Aging, as the main risk factor for most cancer types, captures the effects of cumulative exposure to exogenous and endogenous risk factors in one’s lifetime, and thus the epigenetic drift reflects molecular alterations caused by both genetic and environmental risk factors [21]. Several studies [33,34,35] have investigated whether DNA methylation changes with age were different in males and females, using heterogeneous methods, data sources, and sample sizes. To our knowledge, our study using MCCS data is the largest single study to investigate cancer risk and survival with systematic, genome-wide assessment and replication of age and age-by-sex methylation signals. We found 32,659, 23,141 and 48 CpG sites at which methylation was strongly and consistently associated with age, sex and age-by-sex, respectively. These associations were all replicated internally, and their replication rate in the external data of GS [33] was as high as 94%, 86% and 91%, respectively. Our results for age-by-sex interaction (4 autosomal and 44 X-linked CpGs) corroborate the findings by McCartney et al. [33] that differences in age-associated DNA methylation between sexes are not frequent except for the X chromosome. At the 44 X-linked CpGs with significant age-by-sex interaction, we found notably higher methylation in females than in males (Figure S1). In these X-linked CpGs, females showed consistent methylation levels at ~50% (methylation M-values ~0) whereas males showed on average notably lower and more variable methylation. This observation is consistent with X-chromosome inactivation (XCI) associated with the dosage compensation of two X chromosomes in females that is stable over time [36]. Interestingly, males still showed strong epigenetic drift at these CpGs. For the 24 genes mapping to the 44 X-linked CpGs (Table S4), we queried them against a list of 114 robustly described XCI-escape genes [37] and found that *FLNA*, *IDS*, *IGBP1* and *MID1IP1* were among the 114 XCI escapees.

Our results suggest that DNA methylation at several individual age-related CpGs is negatively associated with risk of cancer overall and mature B-cell lymphoma (Table 3). In the current study, DNA was extracted from pre-diagnostic peripheral blood, which may explain why comparatively more associations were observed for B-cell lymphoma compared with other types of cancer. Among these CpGs (Table 3), DNA methylation at cg25119261 (*HLA-DPB2*) was reported to be differentially methylated between tumor and matched adjacent normal tissues in the context of oral squamous cell [38] and hepatocellular cancer [39]. These conclusions may corroborate our finding of overall cancer association at this site, although we used DNA methylation from pre-diagnostic blood. The CpG cg26738106 (*MXRA5*) was negatively associated with risk of colorectal cancer in our data, but the age-by-sex interaction observed in the set of controls was not replicated in the set of cases and therefore requires further investigation. It is, however, interesting to note that *MXRA5* was shown to be aberrantly expressed in colorectal tumor tissue [40]. A study using exome sequencing has also identified *MXRA5* as a cancer gene frequently mutated in non-small cell lung carcinoma [41], and cg26738106 in *MXRA5* was also nominally significantly associated (*p* < 0.05) with survival from lung cancer in our data: HR = 1.59 (95% CI: 1.17–2.15), *p* = 0.003 in Model 4.1 and HR = 1.58 (95% CI: 1.16–2.14), *p* = 0.004 in Model 4.2.

One age-by-sex-associated CpG cg12054453 in *TMEM49* (also known as *VMP1*) was negatively associated with overall cancer survival. *TMEM49* has been widely reported as a cancer-relevant cell cycle modulator and its expression regulates the invasion and metastatic potential of cancer cells [42,43,44]. *ARX* is a homeobox-containing gene which is expressed primarily in the central and/or peripheral nervous system. Our study suggests that blood DNA methylation at cg21729122, located in the TSS1500 region of *ARX,* may be associated with sex-specific survival from colorectal cancer. Although these findings should be interpreted with caution given methylation was measured in different tissues, they altogether suggest potential for blood DNA methylation in this region to provide a sex-specific mode of early detection and prediction of cancer.

Our results also showed a positive trend in the association between epigenetic drift and risk of cancer overall (slope = 0.17) and cancers of the kidney (slope = 0.04), lung (slope = 0.13), and urothelium (slope = 0.33) and mature B-cell lymphoma (slope = 0.88), as well as a negative trend for gastric (slope = −0.01), prostate (slope = −0.12) and colorectal (slope = −0.09) cancers (Figure 3). This indicates that while our study may have been underpowered to detect associations at individual CpG sites, there is a strong link between aging of the blood methylome and risk of cancer. In two recent studies [28,29], we investigated, using the same samples, the association between cancer risk and several synthetic measures of epigenetic aging. Most associations were overall similar but stronger for the second-generation measures such as *PhenoAge*, a composite biomarker of mortality, and *GrimAge*, a predictor of lifespan generated using several DNA methylation surrogates for plasma proteins and smoking history [24]. For example, for *GrimAge*, we found that associations were in the same direction as the epigenetic drift (current results) with risk of most cancer types: overall (OR = 1.11), kidney (OR = 1.28), lung (OR = 2.03), urothelial (OR = 1.22), gastric (OR = 0.95), prostate (OR = 0.84) cancers and mature B-cell lymphoma (OR = 1.03) [29]. We observed, however, that for colorectal cancer, the association of epigenetic drift with cancer risk was in opposite direction as with *GrimAge* (OR = 1.12), *PhenoAge* (OR = 1.18) [29] and “first-generation” epigenetic clocks [28]. This finding requires further investigation but could reflect biological differences between epigenetic drift and epigenetic clocks [21]. For prostate cancer, the strong negative tendency might reflect the advantaged background of men widely diagnosed with the disease via increased surveillance and testing in Australia.

There are several limitations in this study. First, there might have been a small proportion of closely related individuals between discovery and replication samples, which may result in some inflation of replicated signals. The fact that controls and cases were sampled from the same population and were all processed at the same time and using the same normalization pipeline may also result in somewhat overconfident replication, compared with completely independent samples. Assessing associations using the set of cases may also result in collider bias, but it would likely be small in our setting given the matching of cases and controls [45,46]. Secondly, the external GS data used the EPIC methylation array, which does not capture all the CpGs in our study using HM450; nevertheless, the vast majority of signals were replicated, so we anticipate that methylation sites that did not overlap would show similar replication rates. Despite these potential issues, our replication strategy was overall very stringent, as it used twice the Bonferroni correction for multiple testing, and showed very high consistency of the CpGs associated with age, sex, and age-by-sex. Thirdly, the sample sizes for investigating risk of and survival from specific cancers might not have been sufficiently large (only hundreds of each), to estimate associations with very good precision. It is, therefore, possible that associations were not detected by our study, but these would presumably be relatively weak and could be established via pooling our results with other methylation studies with similar design. Although little evidence of associations was found in the analyses of cancer risk and survival overall (all types), these analyses grouped together distinct cancer types, so might not be biologically relevant for all CpGs. Finally, we could not include a replication phase for the analyses of cancer risk and survival, so our findings of potential cancer biomarkers should be replicated in other studies, and mechanistic studies should be undertaken.

## 4. Materials and Methods

### 4.1. Study Sample

The Melbourne Collaborative Cohort Study (MCCS) is an Australian prospective cohort study of 41,513 people of white European descent recruited between 1990 and 1994 in Melbourne metropolitan area. DNA was extracted from pre-diagnostic peripheral blood taken at recruitment (1990–1994) or at a subsequent follow-up visit (2003–2007) in cancer-free participants. More details about the cohort profile, blood collection, DNA extraction and cancer ascertainment can be found elsewhere [47]. Here we used a total of 3215 case–control pairs from seven specific studies of colorectal (*N* = 835), gastric (*N* = 170), kidney (*N* = 143), lung (*N* = 332), prostate (*N* = 869) and urothelial (*N* = 428) cancers, and mature B-cell lymphoma (*N* = 438) nested within the MCCS. Cases and controls were matched on age at blood draw, sex, country of birth (Australia/New Zealand, Greece, Italy, or United Kingdom/other) and sample type (peripheral blood mononuclear cells, dried blood spots or buffy coats) using incidence density sampling. To minimize batch effects, samples from each matched case–control pair were plated to adjacent wells on the same BeadChip microarray, with plate, chip and position assigned randomly. Case–control pairs with missing values for the confounders (smoking status (current/former/never) and pack-years (log-transformed), alcohol consumption in the previous week (in grams/day) and body mass index (in kg/m^2^)) were excluded. 

### 4.2. Quality Control of DNA Methylation Data

DNA methylation in the MCCS was measured using the Illumina HumanMethylation450 (HM450) assay (Illumina, Inc., San Diego, CA, USA). Quality control (QC) details for processing methylation beta values have been reported previously [28]. Briefly, we removed CpGs with missing rate > 20% based on the sample and CpGs on Y-chromosome. M-values, calculated as log_2_ (beta/(1 − beta)), were then used for the analyses since these are thought to be more statistically valid for detection of differential methylation [48].

### 4.3. Statistical Analyses

#### 4.3.1. Discovery and Replication Sets

To identify age-, sex- and age-by-sex-related methylation markers (Aims 1 and 2), we used all control subjects from the MCCS sample as a discovery set. After excluding duplicated controls across the seven studies (an individual may be assigned as a control in several different studies), 3008 participants were available for the discovery phase analysis. After QC of methylation data on the discovery sample, there remained 484,828 available CpGs. All study cases were then used as the replication set. After excluding duplicates across the seven studies (an individual may be diagnosed with more than one specific cancer type and thus was selected as a case in several studies; in such instances, we included the first diagnosis only) and participants with samples that overlapped with the discovery set (an individual with a specific cancer may be assigned as a control in a different study), 2754 participants were available for the replication phase analysis.

McCartney et al. [33] recently studied age-, sex- and age-by-sex-associated CpGs using DNA methylation data of Illumina EPIC array (Illumina, Inc., San Diego, CA, USA) and samples (2586 individuals for discovery and 4450 individuals for replication) from Generation Scotland (GS)—Scottish Family Health Study. We used their summary statistics [49] as an external replication set.

#### 4.3.2. Age, Sex, and Age-By-Sex Associations

To assess associations of age and sex with DNA methylation (Aim 1), we conducted epigenome-wide association studies (EWAS). We fitted linear mixed regression models of methylation M-values at individual CpGs on age, sex, country of birth and sample type as fixed effect variables, and study of specific cancer type, assay plate and slide as random effect variables. A first model (Model 1.1) was adjusted for white blood cell composition (percentage of CD4 + T cells, CD8 + T cells, B cells, NK cells, monocytes and granulocytes, estimated using the Houseman algorithm [50]), and a second model (Model 1.2) was additionally adjusted for lifestyle factors (smoking status and pack-years, alcohol consumption in the previous week and BMI [51,52,53]). Age-associated and sex-associated changes in DNA methylation were then assessed by examining regression coefficients for the variables age and sex, respectively.

(1) M_value ~ age+sex+country_of_birth+sample_type+CD4T+CD8T+Bcell+NK+Mono+Gran+1|study+1|plate_level+1|plate_slide

(Model 1.1)

(2) M_value ~ age+sex+country_of_birth+sample_type+CD4T+CD8T+Bcell+NK+Mono+Gran+smoking_status+pack_years+alcohol+BMI+1|study+1|plate_level+1|plate_slide

(Model 1.2)

To investigate sex-specific ageing-associated changes in DNA methylation (Aim 2), we added an interaction term age × sex to the same models (Model 2.1 and Model 2.2).

(3) M_value ~ age+sex+age×sex+country_of_birth+sample_type+CD4T+CD8T+Bcell+NK+Mono+Gran+1|study+1|plate_level+1|plate_slide

(Model 2.1)

(4) M_value ~ age+sex+age×sex+country_of_birth+sample_type+CD4T+CD8T+Bcell+NK+Mono+Gran+smoking_status+pack_years+alcohol+BMI+1|study+1|plate_level+1|plate_slide

(Model 2.2).

In the discovery phase, we used the Bonferroni correction for multiple testing to declare statistical significance (*p* < 0.05/484,828 = 1.0 × 10^−7^) for all Model 1s and 2s. The same models were then carried out using the replication set. Replicated findings were further examined using the GS summary statistics [33,49] (considering only CpGs common to the HM450 and EPIC assays). The replication criteria included multiple comparisons (*p*-value threshold for replication was based on Bonferroni correction) and same effect directions. A flowchart detailing the study design and analysis pipeline was presented in Figure 1.

#### 4.3.3. Cancer Risk and Survival Associations

We assessed associations between DNA methylation at individual age- and age-by-sex-associated CpGs and risk of overall cancer (using the 2754 matched case–control pairs with no duplicated participant involved) and cancer at seven specific sites, using conditional logistic regression models (Models 3.1 and 3.2) to estimate odds ratio (OR) and 95% confidence intervals (CI), expressed per standard deviation (SD). In Model 3.1, we adjusted for age, country of birth and white blood cell composition. Sex and sample type were exactly matched between cases and controls so were not adjusted for. Model 3.2 was additionally adjusted for smoking status (current/former/never) and pack-years, alcohol consumption in the previous week (grams/day, continuous) and BMI (continuous). The methylation sites at which associations were significant after Bonferroni correction for multiple comparisons in both Models 3.1 and 3.2 were considered to be associated with risk of cancer.

We used Cox models (Models 4.1 and 4.2) to estimate hazard ratios (HR) per SD for the associations between M-values at individual age- and age-by-sex-associated CpG sites and risk of death (all causes) following cancer diagnosis. The survival analysis was thus restricted to cancer cases. A total of *n* = 1931 deaths were included in the analysis. Time since diagnosis was used as the timescale, and person-years of follow-up were calculated from the diagnosis date until the date of death, and censored at the date of departure from Australia or end of follow-up. Where a participant was diagnosed with several cancers, we considered only the first diagnosis to count follow-up time. Number of deaths by cancer type was as follows: colorectal cancer: *n* = 526, gastric cancer: *n* = 144, kidney cancer: *n* = 80, lung cancer: *N* = 311, prostate cancer: *n* = 435, urothelial cancer: *n* = 234, and mature B-cell lymphoma: *n* = 286. In Model 4.1, we adjusted for age, sex, country of birth, sample type and white blood cell composition. Model 4.2 was additionally adjusted for smoking status and pack-years, alcohol consumption and BMI. The methylation sites at which associations were Bonferroni-significant in both Models 4.1 and 4.2 were considered to be associated with cancer survival. All statistical analyses were performed using R version 3.6.0.

## 5. Conclusions

Our study is the first large-scale study to examine potential genes at which blood DNA methylation is age-related (including modification by sex) and associated with cancer development and progression. Significant associations with cancer risk and survival were identified at several individual age-related CpG sites. We also observed a strong negative trend in the association between epigenetic drift and risk of colorectal cancer, i.e., in opposite direction to our previous findings using epigenetic clocks. Two CpGs at which the effect of age on DNA methylation was different in males and females (at the *TMEM49* and *ARX* loci) were associated with survival of all cancers and colorectal cancer, respectively. Our results could be useful for developing strategies of early diagnosis of cancer and prediction of cancer prognosis. Additional studies are required to replicate our findings and identify the potential mechanisms by which epigenetic drift is associated with cancer risk and survival.

## Figures and Tables

**Table 1:** Characteristics of the participants in the MCCS sample included in the analysis.

Characteristics	MCCS Sample	
	Cancer Cases (*n* = 2754)	Controls (*n* = 3008)
Age at blood draw, median (IQR)	61.1	61.1
	(54.2–66.0)	(54.3–65.8)
Sex:		
male, *n* (%)	1846 (67%)	2067 (68.7%)
female, *n* (%)	908 (33%)	941 (31.3%)
Country of birth:		
Australia/New Zealand, *N* (%)	1847 (67.1%)	2011 (66.9%)
Greece, *n* (%)	291 (10.6%)	322 (10.7%)
Italy, *n* (%)	430 (15.6%)	478 (15.9%)
UK/other, *n* (%)	186 (6.8%)	197 (6.5%)
Blood sample type:		
dried blood spots, *n* (%)	1880 (68.3%)	2053 (68.3%)
peripheral blood mononuclear cells, *N* (%)	706 (25.6%)	767 (25.5%)
buffy coats, *n* (%)	168 (6.1%)	188 (6.3%)
Smoking status:		
current, *n* (%)	417 (15.1%)	429 (14.3%)
former, *n* (%)	1128 (41%)	1198 (39.8%)
never, *n* (%)	1209 (43.9%)	1381 (45.9%)
Smoking pack-years, median (IQR)	4	2.4
	(0–30.1)	(0–27)
Body mass index (kg/m^2^), median (IQR)	26.9	26.8
	(24.5–29.7)	(24.5–29.4)
Alcohol consumption (g/day), median (IQR)	5.2	4.3
	(0–19)	(0–19)

Note: MCCS: Melbourne Collaborative Cohort Study. IQR: interquartile range.

**Table 2:** Thirty age-by-sex associations common to discovery (*p* < 1.0 × 10^−7^) and replication (*p* < 6.9 × 10^−4^) sets in MCCS and also replicated using GS summary statistics.

CpG	CHR	MAPINFO	Strand	Gene	Gene Feature	Discovery Set in MCCS			Replication Set in MCCS		
						Effect	SE	*p*-Value	Effect	SE	*p*-Value
cg12054453	17	57915717	F	*TMEM49*	Body	0.22	0.03	1.81 × 10^−12^	0.18	0.03	5.56 × 10^−8^
cg06758848	X	153603268	F	*FLNA*	TSS1500	0.14	0.02	9.77 × 10^−15^	0.16	0.02	3.41 × 10^−16^
cg13775533	X	99663073	F	*PCDH19*	1stExon	−0.14	0.02	2.55 × 10^−14^	−0.09	0.02	8.69 × 10^−7^
cg09761247	X	148585951	R	*IDS*	Body	0.23	0.03	6.57 × 10^−13^	0.14	0.03	2.96 × 10^−5^
cg08783090	X	151143125	R	*GABRE*	1stExon	−0.12	0.02	7.85 × 10^−13^	−0.08	0.02	3.27 × 10^−6^
cg25888700	X	21676344	R	*KLHL34*	5′UTR	−0.11	0.02	2.96 × 10^−12^	−0.08	0.02	3.68 × 10^−7^
cg01828474	X	21676593	R	*KLHL34*	TSS200	−0.11	0.02	8.17 × 10^−12^	−0.09	0.02	1.45 × 10^−7^
cg17036062	X	25034037	F	*ARX*	1stExon	−0.09	0.01	9.43 × 10^−12^	−0.06	0.01	1.20 × 10^−5^
cg25988710	X	72667379	R	*CDX4*	1stExon	−0.11	0.02	2.47 × 10^−11^	−0.07	0.02	3.18 × 10^−5^
cg06775759	X	21676994	R	*KLHL34*	TSS1500	−0.10	0.02	4.24 × 10^−11^	−0.09	0.02	2.92 × 10^−8^
cg03671371	X	36975540	R	*-*	*-*	−0.11	0.02	4.72 × 10^−11^	−0.09	0.02	1.34 × 10^−6^
cg08814148	X	118407645	F	*-*	*-*	−0.14	0.02	1.86 × 10^−10^	−0.14	0.02	1.57 × 10^−10^
cg16108684	X	125300035	F	*DCAF12L2*	TSS200	−0.12	0.02	3.66 × 10^−10^	−0.08	0.02	4.02 × 10^−5^
cg24823082	X	38660587	F	*MID1IP1*	TSS200	−0.15	0.02	4.31 × 10^−10^	−0.09	0.03	2.79 × 10^−4^
cg25156485	X	64887827	R	*MSN*	Body	0.08	0.01	5.02 × 10^−10^	0.08	0.01	1.97 × 10^−10^
cg04424215	X	111325143	R	*TRPC5*	5′UTR	−0.13	0.02	6.53 × 10^−10^	−0.08	0.02	1.18 × 10^−4^
cg25528646	X	151143302	R	*GABRE*	TSS200	−0.09	0.01	8.18 × 10^−10^	−0.06	0.02	4.62 × 10^−5^
cg24931094	X	119737891	R	*MCTS1*	5′UTR	0.09	0.01	1.31 × 10^−9^	0.07	0.01	1.12 × 10^−6^
cg12537796	X	106515818	R	*-*	*-*	−0.12	0.02	1.86 × 10^−9^	−0.09	0.02	8.22 × 10^−6^
cg25127732	X	21676692	F	*KLHL34*	TSS1500	−0.08	0.01	2.71 × 10^−9^	−0.05	0.01	3.56 × 10^−4^
cg03202526	X	139587311	F	*SOX3*	TSS200	−0.07	0.01	3.62 × 10^−9^	−0.05	0.01	1.52 × 10^−5^
cg11194545	X	105066793	F	*NRK*	5′UTR	−0.10	0.02	4.06 × 10^−9^	−0.08	0.02	9.14 × 10^−7^
cg06779802	X	107979401	F	*IRS4*	1stExon	−0.08	0.01	7.73 × 10^−9^	−0.07	0.01	1.30 × 10^−6^
cg22606540	X	100914483	R	*ARMCX2*	5′UTR	−0.10	0.02	1.23 × 10^−8^	−0.07	0.02	3.86 × 10^−5^
cg21729122	X	25034488	F	*ARX*	TSS1500	−0.10	0.02	1.34 × 10^−8^	−0.08	0.02	1.55 × 10^−5^
cg02295369	X	117959263	R	*ZCCHC12*	Body	−0.09	0.02	3.41 × 10^−8^	−0.06	0.02	2.28 × 10^−4^
cg23208903	X	30327487	F	*NR0B1*	5′UTR	−0.09	0.02	3.82 × 10^−8^	−0.06	0.02	2.10 × 10^−4^
cg14586560	X	133118453	R	*GPC3*	Body	−0.08	0.01	4.43 × 10^−8^	−0.06	0.01	8.19 × 10^−5^
cg21040569	X	137793665	F	*FGF13*	Body	−0.08	0.02	5.24 × 10^−8^	−0.05	0.02	5.55 × 10^−4^
cg09407917	X	130192151	R	*FLJ30058*	TSS200	−0.09	0.02	6.52 × 10^−8^	−0.07	0.02	2.52 × 10^−5^

**Table 3:** CpGs at which methylation was associated with age and with risk of cancer.

CpG	CHR	MAPINFO	Strand	Gene	Gene Feature	Model 3.1		Model 3.2		Specific Cancer
						OR (95%CI)	*p*-Value	OR (95%CI)	*p*-Value	
cg25119261	6	33081351	R	*HLA-DPB2*	Body	0.80 (0.73–0.87)	1.63 × 10^−7^	0.80 (0.73–0.87)	1.52 × 10^−7^	Overall
cg05497216	16	89408076	R	*ANKRD11*	5′UTR	0.83 (0.78–0.89)	3.04 × 10^−7^	0.83 (0.78–0.89)	4.94 × 10^−7^	
cg05772125	16	31539169	R	*AHSP*	TSS200	0.40 (0.29–0.57)	2.20 × 10^−7^	0.39 (0.27–0.55)	1.59 × 10^−7^	Mature B-cell lymphoma
cg04771285	12	117557630	R	*-*	*-*	0.49 (0.37–0.64)	3.28 × 10^−7^	0.49 (0.37–0.65)	6.49 × 10^−7^	
cg09046979	16	28333134	R	*SBK1*	3′UTR	0.61 (0.50–0.74)	7.92 × 10^−7^	0.60 (0.49–0.73)	4.80 × 10^−7^	
cg11876705	19	38918253	R	*RASGRP4*	TSS1500	0.44 (0.32–0.61)	9.00 × 10^−7^	0.45 (0.32–0.62)	1.44 × 10^−6^	
cg06774893	16	68011109	R	*DPEP3*	Body	0.46 (0.34–0.63)	1.26 × 10^−6^	0.43 (0.31–0.59)	2.55 × 10^−7^	
cg22361106	1	33909498	R	*-*	*-*	0.46 (0.34–0.63)	1.31 × 10^−6^	0.44 (0.32–0.61)	7.69 × 10^−7^	

Note: Conditional logistic regression Models 3.1 and 3.2 were used to estimate OR and 95% CI per standard deviation. In Model 3.1, we adjusted for age, country of birth and white blood cell composition as sex and sample type were exactly matched between cases and controls. Model 3.2 was additionally adjusted for lifestyle confounders of smoking status and pack-years, alcohol consumption in the previous week and BMI. The significance level for age-related methylation association with cancer risk is *p* = 1.5 × 10^−6^.

**Table 4:** Top 20 CpGs associated with age and with survival from cancer overall.

CpG	CHR	MAPINFO	Strand	Gene	Gene Feature	Model 4.1		Model 4.2	
						HR (95%CI)	*p* Value	HR (95%CI)	*p* Value
cg26427498	7	105987258	R	*-*	*-*	0.78 (0.74–0.82)	8.35 × 10^−19^	0.80 (0.76–0.85)	1.21 × 10^−14^
cg26470501	19	45252955	F	*BCL3*	Body	0.80 (0.76–0.84)	6.87 × 10^−18^	0.84 (0.80–0.89)	6.68 × 10^−11^
cg25143652	20	62168670	R	*PTK6*	1stExon	0.76 (0.72–0.81)	1.56 × 10^−16^	0.81 (0.76–0.87)	3.20 × 10^−10^
cg01127300	22	38614796	F	*-*	*-*	0.82 (0.78–0.86)	5.19 × 10^−16^	0.87 (0.83–0.91)	1.07 × 10^−8^
cg08857221	1	37941361	R	*ZC3H12A*	Body	0.76 (0.71–0.81)	1.34 × 10^−15^	0.78 (0.73–0.84)	4.82 × 10^−11^
cg19572487	17	38476024	R	*RARA*	5′UTR	0.81 (0.77–0.86)	4.51 × 10^−14^	0.87 (0.82–0.92)	4.85 × 10^−7^
cg02773019	3	135684688	F	*PPP2R3A*	5′UTR	1.21 (1.15–1.27)	6.22 × 10^−14^	1.20 (1.14–1.26)	7.34 × 10^−13^
cg15114651	19	47289410	F	*SLC1A5*	TSS1500	0.77 (0.72–0.83)	7.16 × 10^−14^	0.83 (0.78–0.89)	2.30 × 10^−7^
cg07069636	16	30671749	F	*-*	*-*	0.79 (0.74–0.84)	1.04 × 10^−13^	0.85 (0.79–0.90)	2.95 × 10^−7^
cg15962267	5	138612986	F	*SNHG4*	Body	0.77 (0.72–0.83)	1.54 × 10^−13^	0.81 (0.75–0.87)	3.70 × 10^−8^
cg02584867	19	1861099	R	*KLF16*	Body	0.83 (0.79–0.87)	1.56 × 10^−13^	0.85 (0.81–0.89)	5.84 × 10^−11^
cg09287933	6	33384473	R	*CUTA*	Body	0.75 (0.70–0.81)	1.56 × 10^−13^	0.77 (0.72–0.84)	8.99 × 10^−11^
cg03519879	14	74227499	R	*C14orf43*	5′UTR	0.77 (0.72–0.83)	2.46 × 10^−13^	0.82 (0.77–0.88)	3.72 × 10^−8^
cg12170787	19	1130965	R	*SBNO2*	Body	0.76 (0.71–0.82)	5.32 × 10^−13^	0.80 (0.74–0.86)	5.26 × 10^−9^
cg07148697	6	31323253	R	*HLA-B*	Body	0.76 (0.71–0.82)	5.67 × 10^−13^	0.77 (0.72–0.84)	3.32 × 10^−11^
cg26283141	6	33240471	F	*VPS52*	TSS1500	0.82 (0.77–0.86)	9.04 × 10^−13^	0.84 (0.79–0.89)	9.42 × 10^−10^
cg25264101	19	14064374	F	*PODNL1*	TSS200	0.84 (0.80–0.88)	1.29 × 10^−12^	0.86 (0.82–0.90)	3.27 × 10^−10^
cg05352838	6	33384391	R	*CUTA*	3′UTR	0.73 (0.67–0.80)	2.44 × 10^−12^	0.76 (0.69–0.83)	1.48 × 10^−9^
cg19513004	6	34206683	F	*HMGA1*	5′UTR	0.80 (0.75–0.85)	2.70 × 10^−12^	0.83 (0.78–0.88)	2.68 × 10^−9^
cg02003183	14	103415882	F	*CDC42BPB*	Body	1.21 (1.14–1.27)	3.43 × 10^−12^	1.17 (1.11–1.23)	8.61 × 10^−9^

Note: Cox regression Models 4.1 and 4.2 were used to estimate OR and 95% CI per standard deviation. In Model 4.1, we adjusted for age, sex, country of birth, sample type and white blood cell composition. Model 4.2 was additionally adjusted for smoking status and pack-years, alcohol consumption and BMI. The significance level for age-related methylation association with cancer risk is *p* = 1.5 × 10^−6^.

## Data Availability

Data will be made available upon reasonable request to the corresponding author.

## Supplementary Materials

The following are available online at https://www.mdpi.com/article/10.3390/cancers13081881/s1, Figure S1: Associations of methylation M-values with age-by-sex for the 48 CpGs. Table S1. Characteristics of the participants from seven specific studies in the MCCS sample. Table S2: 32,659 replicated age-associated CpGs using the MCCS sample. Table S3: 23,141 replicated sex-associated CpGs using the MCCS sample. Table S4: 48 replicated age-by-sex-associated CpGs using the MCCS sample (*p* < 1.03 × 10^−7^ for controls and *p* < 6.85 × 10^−4^ for cases), Table S5: 126 (all autosomal) age-related CpGs were associated with survival of cancer overall. Table S6: Significant age-related CpGs with survival of gastric cancer (30 autosomal sites), lung cancer (282 autosomal and 1 X-linked) and mature B-cell lymphoma (1 autosomal site).
