# Supplementary material for: Epigenetic Drift Association with Cancer Risk and Survival, and Modification by Sex
Source: Cancers (Basel). 2021 Apr 14;13(8):1881. doi: 10.3390/cancers13081881 (PMC8070898; doi:10.3390/cancers13081881)
Supplement: Supplementary file 1 [file cancers-13-01881-s001.zip › cancers-1166912-supplementary.pdf]

# Supplementary Materials: Epigenetic Drift Association with Cancer Risk and Survival, and Modification by Sex

**Chenglong Yu, Ee Ming Wong, Jihoon Eric Joo, Allison M. Hodge, Enes Makalic, Daniel Schmidt, Daniel D. Buchanan, Gianluca Severi, John L. Hopper, Dallas R. English, Graham G. Giles, Melissa C. Southey and Pierre-Antoine Dugué**

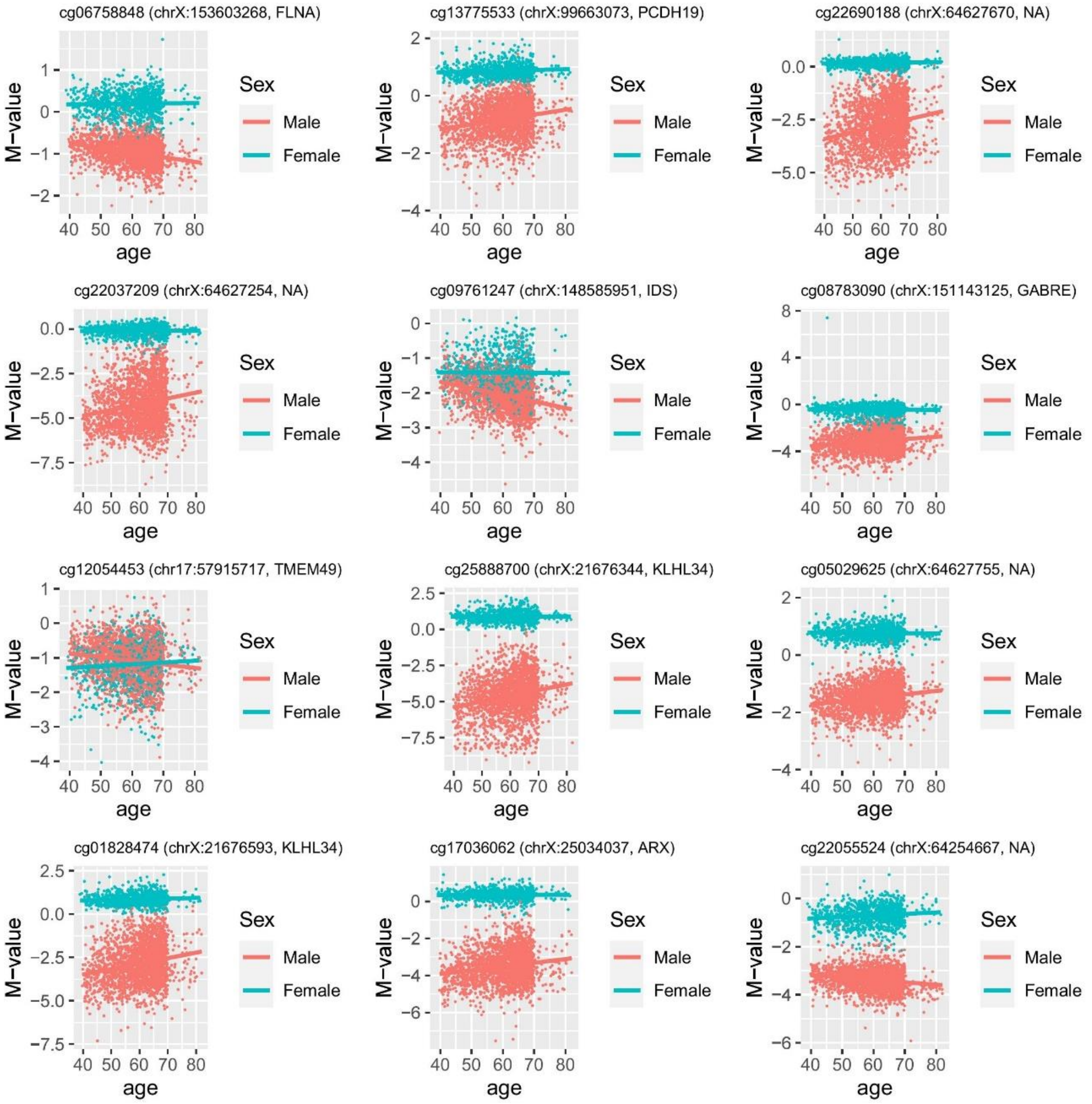

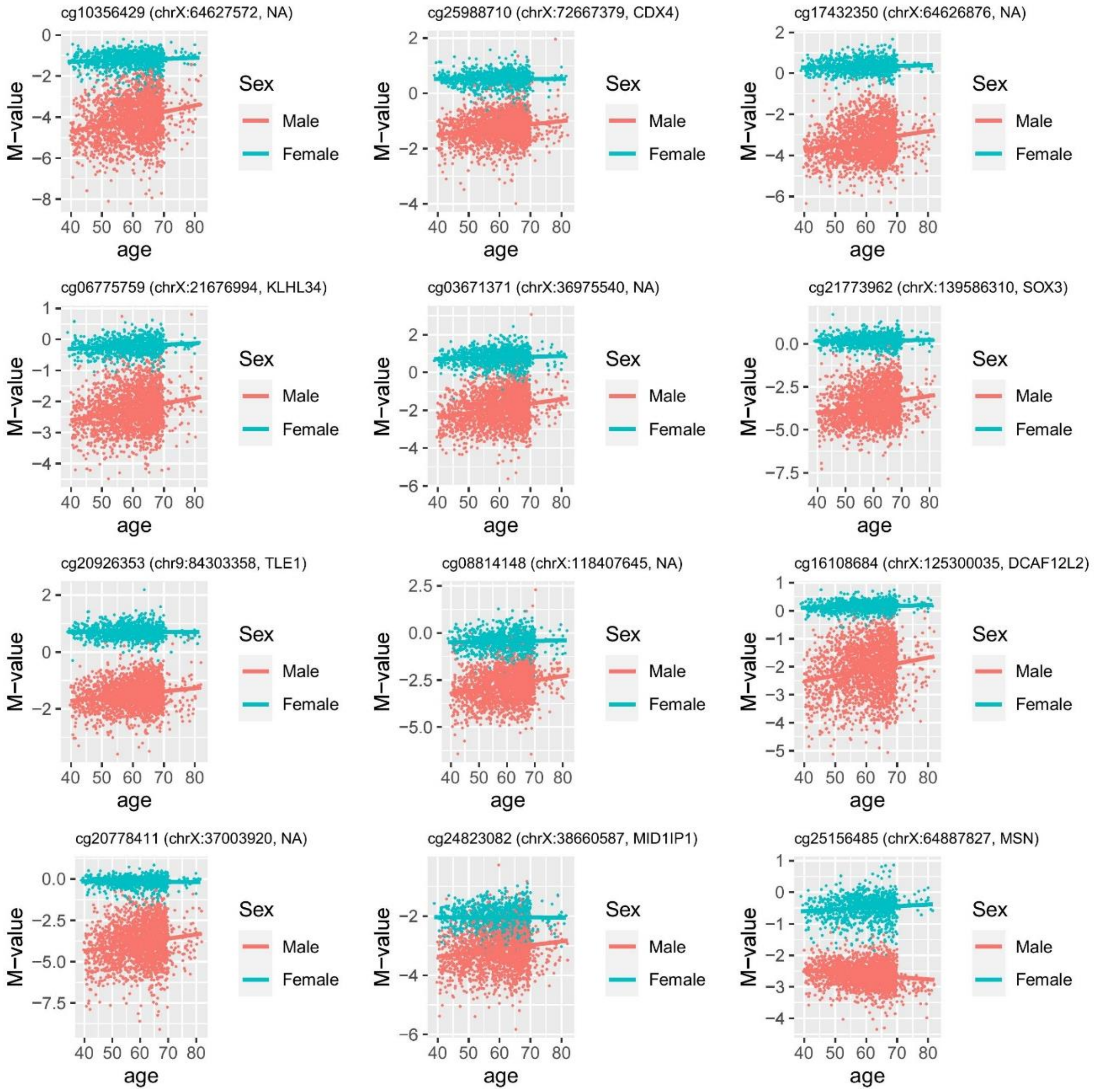
cg10356429 (chrX:64627572, NA)
cg25988710 (chrX:72667379, CDX4)
cg17432350 (chrX:64626876, NA)
cg06775759 (chrX:21676994, KLHL34)
cg03671371 (chrX:36975540, NA)
cg21773962 (chrX:139586310, SOX3)
cg20926353 (chr9:84303358, TLE1)
cg08814148 (chrX:118407645, NA)
cg16108684 (chrX:125300035, DCAF12L2)
cg20778411 (chrX:37003920, NA)
cg24823082 (chrX:38660587, MID1IP1)
cg25156485 (chrX:64887827, MSN)
M−value
age
Sex
Male
Female

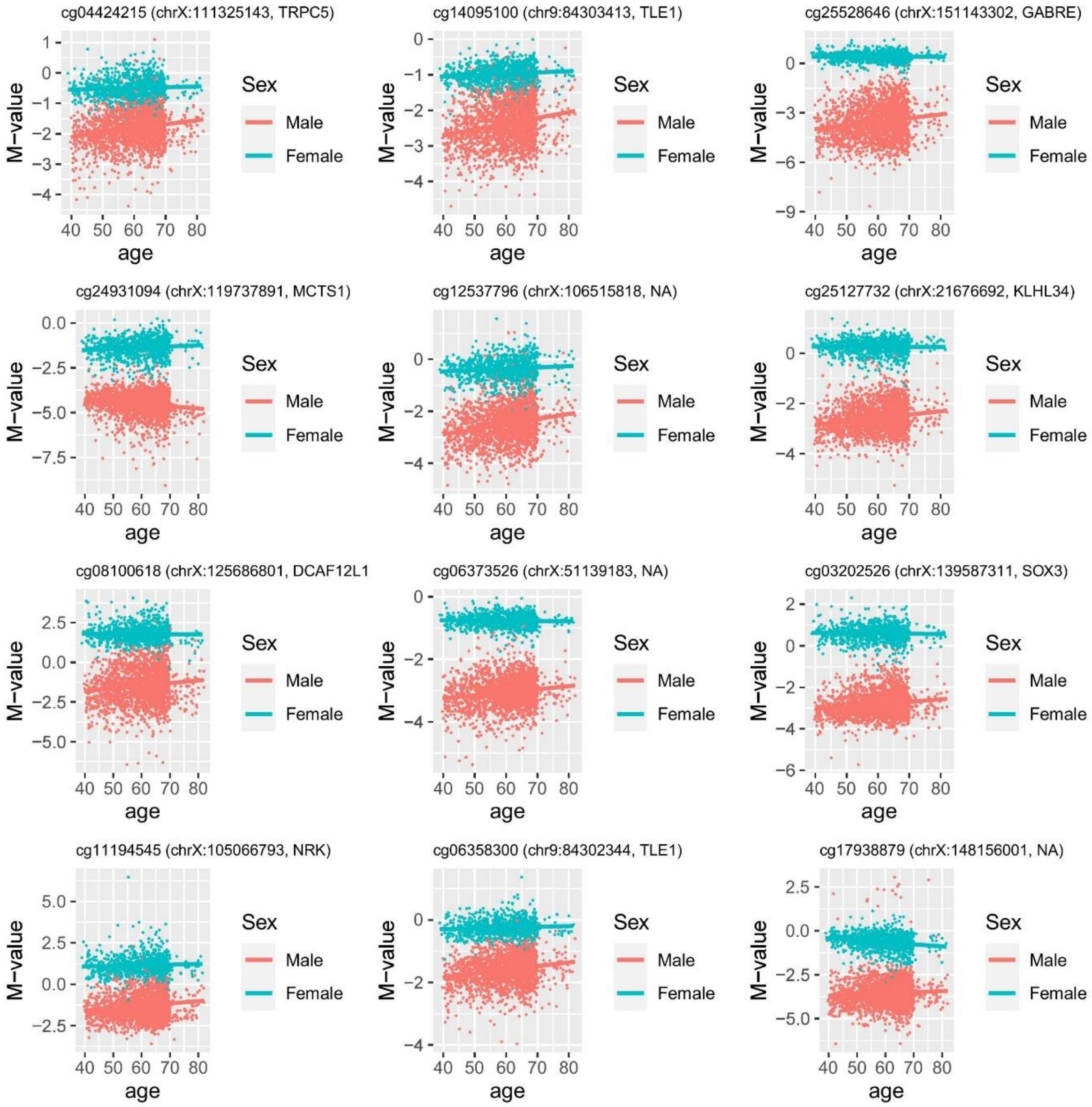
cg04424215 (chrX:111325143, TRPC5)
cg14095100 (chr9:84303413, TLE1)
cg25528646 (chrX:151143302, GABRE)
cg24931094 (chrX:119737891, MCTS1)
cg12537796 (chrX:106515818, NA)
cg25127732 (chrX:21676692, KLHL34)
cg08100618 (chrX:125686801, DCAF12L1
cg06373526 (chrX:51139183, NA)
cg03202526 (chrX:139587311, SOX3)
cg11194545 (chrX:105066793, NRK)
cg06358300 (chr9:84302344, TLE1)
cg17938879 (chrX:148156001, NA)
M−value
age
Sex
Male
Female

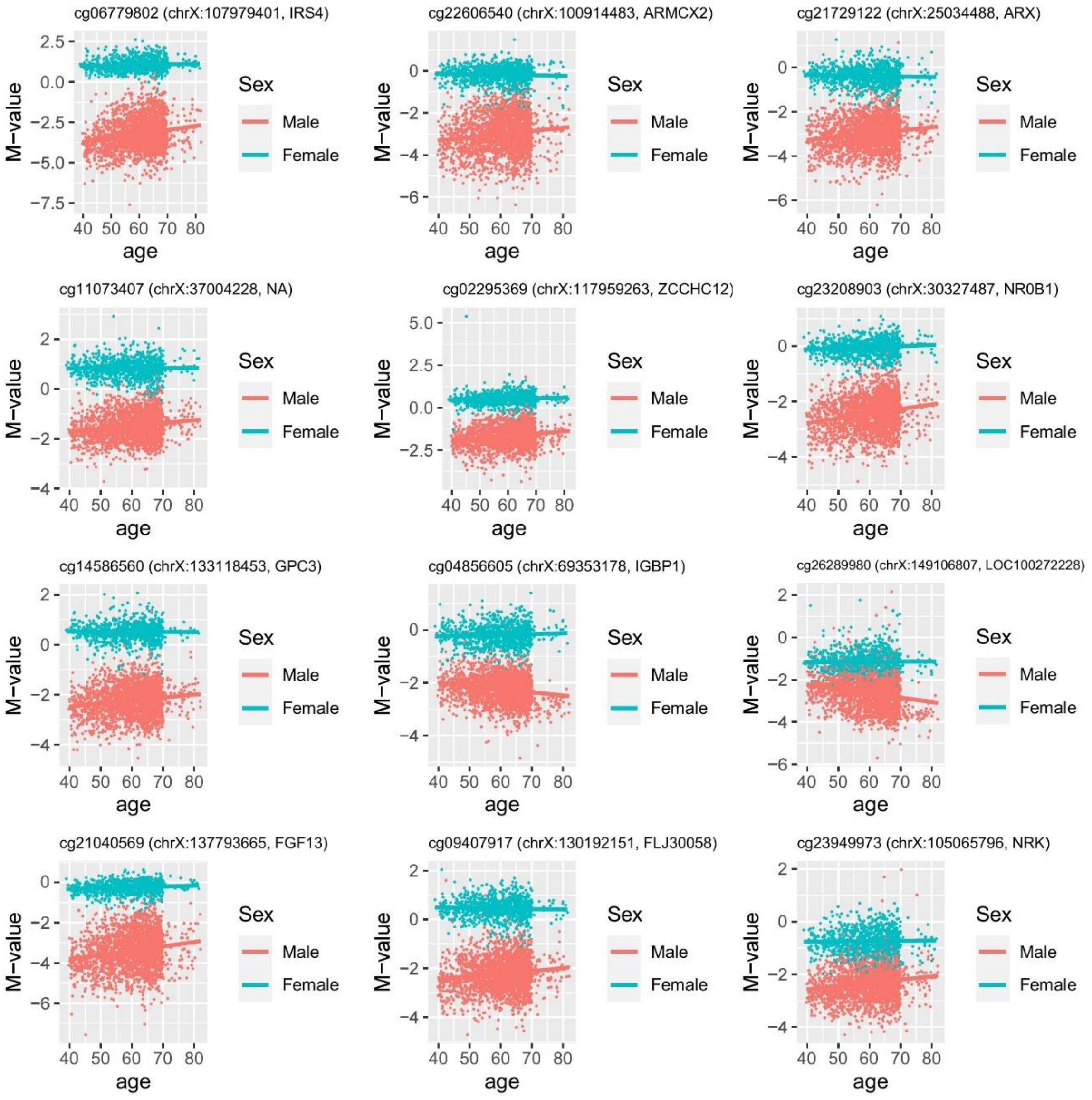


**Figure S1.** Associations of methylation M-values with age-by-sex for the 48 CpGs.

**Table S1.** Characteristics of the participants from seven specific studies in the MCCS sample.

**Table S2.** 32,659 replicated age-associated CpGs using the MCCS sample.

**Table S3.** 23,141 replicated sex-associated CpGs using the MCCS sample.

**Table S4.** 48 replicated age-by-sex-associated CpGs using the MCCS sample ($p < 1.03 \times 10^{-7}$ for controls and $p < 6.85 \times 10^{-4}$ for cases). Among them, 30 CpGs were replicated in two GS data ($p < 0.0015$).

**Table S5.** 126 (all autosomal) age-related CpGs were associated with survival of cancer overall.

**Table S6.** Significant age-related CpGs with survival of gastric cancer (30 autosomal sites), lung cancer (282 autosomal and 1 X-linked) and mature B-cell lymphoma (1 autosomal site).

**Table S1–S6.** are provided separately, attached as an Excel File.
